# Mechanistic insights into GPAA1-mediated cold tumor phenotype and immune evasion in colorectal cancer: integrative multi-omics analysis and experimental validation

**DOI:** 10.3389/fonc.2025.1701368

**Published:** 2025-11-24

**Authors:** Jia Zhu, Tao Zhu, Yichen Cai, Hancheng Fan

**Affiliations:** Department of Pathology at Sir Run-Run Shaw Hospital, Zhejiang University School of Medicine, Hangzhou, China

**Keywords:** GPAA1, colorectal cancer, cold tumor, immune evasion, tumor microenvironment

## Abstract

**Background:**

Glycosylphosphatidylinositol(GPI) Anchor Attachment Protein 1 (GPAA1) plays a critical role in GPI-anchor biosynthesis, yet its pan-cancer expression patterns and functional significance in Colorectal Cancer(CRC) remain unclear.

**Methods:**

We integrated multi-omics data (TCGA, GTEx, GEO, HPA) to analyze GPAA1 expression, immune infiltration, and genomic instability in CRC. Single-cell/spatial transcriptomics and functional assays (proliferation, migration, invasion) were performed to validate findings.

**Results:**

GPAA1 was significantly overexpressed in CRC (mRNA and protein, *p* < 0.05) and associated with poor prognosis. High GPAA1 correlated with genomic instability (aneuploidy, HRD) and an immune-cold phenotype (reduced CD8+ T cells, increased M2 macrophages). Functional assays confirmed GPAA1 promotes CRC aggressiveness.

**Conclusion:**

GPAA1 drives CRC progression via genomic instability and immunosuppression, serving as a prognostic biomarker and potential therapeutic target.

## 1 Introduction

CRC is one of the malignant tumors with persistently high incidence and mortality worldwide. Its pathogenesis is complex, and treatment outcomes are constrained by multiple factors such as the tumor microenvironment, genomic instability, and immune status (1–3). In recent years, with the development of pan-cancer analysis and precision medicine technologies, identifying key genes with diagnostic, prognostic value, and therapeutic potential has become a core focus in CRC research.

GPAA1 encodes a key component of the GPI transamidase complex, which is essential for the attachment of GPI anchors to newly synthesized proteins in the endoplasmic reticulum. This post-translational modification enables the anchoring of diverse proteins to the cell membrane, facilitating their participation in critical processes such as signal transduction, cell adhesion, and immune recognition (4). Dysregulation of GPI-anchored proteins has been implicated in various cancers, influencing tumor cell proliferation, metastasis, and interaction with the tumor microenvironment (5, 6). Preliminary evidence suggests that GPAA1 may be overexpressed in certain malignancies, including gastric and hepatocellular carcinomas, and is associated with adverse clinical outcomes. However, a comprehensive pan-cancer analysis of GPAA1, particularly its expression pattern, prognostic value, and mechanistic role in orchestrating the immune landscape of CRC, remains largely unexplored. Elucidating these aspects is crucial for evaluating GPAA1’s efficacy as a prognostic biomarker and its potential as a therapeutic target in CRC. Abnormal expression of genes related to this pathway may drive tumor initiation and progression by regulating tumor cell proliferation, metabolism, and immune microenvironment remodeling (4, 7). However, comprehensive and in-depth research is still lacking on the expression characteristics of GPAA1 in pan-cancers, its association with CRC immune subtypes and genomic instability, as well as its regulatory mechanisms on the malignant phenotype of CRC cells and anti-tumor immune responses.

**Figure 1:**
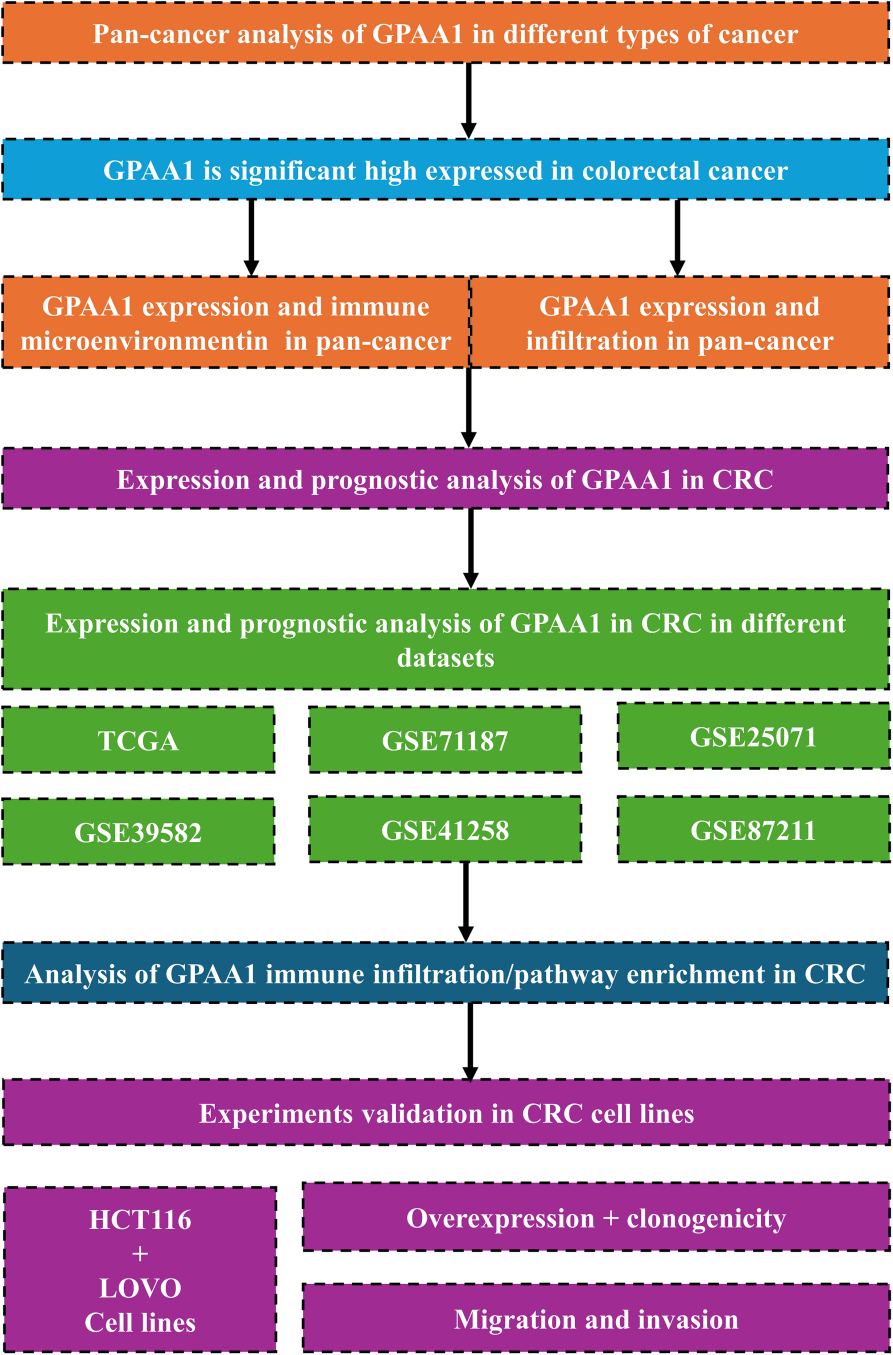
Provides an overview of the study. We first performed a pan-cancer analysis that highlighted the potential role of GPAA1 in colorectal cancer, then interrogated multiple databases to evaluate GPAA1 in CRC, and finally validated the findings using *in vitro* experiments.

Building on this, the present study integrated multiple multi-omics databases, namely TCGA, GTEx, GEO, and HPA. Initially, it examined the expression profiles of GPAA1 across pan-cancers, along with its correlations with immune subtypes and signaling pathways in CRC. Furthermore, by leveraging multi-algorithm immune infiltration analysis and genomic instability correlation analysis, the study explored how GPAA1 regulates the development of the cold tumor phenotype in CRC. In combination with single-cell and spatial transcriptomic techniques, the research clarified the cellular localization of GPAA1 within the local immune microenvironment of CRC, as well as its influence on the composition and spatial distribution of immune cells. Finally, *in vitro* functional experiments—including cell proliferation, colony formation, migration, and invasion assays—were carried out to validate GPAA1’s regulatory role in the malignant biological behaviors of CRC cells. The overall goal of this study is to unravel the function and mechanism of GPAA1 in the initiation and progression of CRC, thereby laying a new theoretical foundation and identifying potential targets for CRC prognostic assessment and targeted therapy. (**Figure 1**).

## 2 Materials and methods

### 2.1 Pan-cancer expression analysis

TPM expression data from TCGA (tcga_RSEM_gene_tpm) and GTEx (gtex_RSEM_gene_tpm) were obtained via UCSC Xena. To mitigate batch effects between TCGA and GTEx datasets, expression data were adjusted using the ComBat function (SVA package). Matched normal tissues were selected from anatomically corresponding GTEx samples, and tumor types with fewer than three normal samples were excluded. Differential expression was evaluated using Wilcoxon Rank Sum tests and visualized via the Sparkle Database.

### 2.2 Pathology analysis

To investigate the protein expression patterns of the GPAA1 gene across various tumor types, we downloaded relevant IHC staining images from the Human Protein Atlas (HPA) database (https://www.proteinatlas.org/). For each tumor type, we recorded the staining intensity and conducted statistical analyses to determine the proportions of samples exhibiting high, medium, low, or undetectable staining levels.

### 2.3 Immune subtype analysis

Based on the median expression level of GPAA1, tumor samples were categorized into high- and low-expression groups. The prevalence of each group across five immune subtypes (C1–C5) was subsequently assessed (8). Significant variations in the distribution of GPAA1 expression among these subtypes were evaluated using Chi-square tests.

### 2.4 Pan-cancer hallmark pathway analysis

In the pan-cancer cohort, samples were stratified based on GPAA1 expression levels: the top 30% were designated as high-expression, and the bottom 30% as low-expression. Differential gene expression analysis was conducted using the limma R package, which computed log_2_ fold changes (Log2FC) for all genes. Genes were subsequently ranked by Log2FC. Hallmark gene set enrichment analysis (GSEA) was then performed using clusterProfiler, calculating enrichment scores (ES) for each gene set. Significance of enrichment was assessed with multiple hypothesis testing, and results were visualized via bubble plots.

### 2.5 Analysis of the immune microenvironment in pan-cancer

In the pan-cancer analysis, we evaluated the correlation between GPAA1 expression and immune cell infiltration using the TIMER2.0 database (http://timer.cistrome.org/), which integrates eight independent immune infiltration estimation algorithms: TIMER, CIBERSORT, CIBERSORT-ABS, QUANTISEQ, XCELL, MCPCOUNTER, EPIC, and TIDE. These algorithms infer the abundance of various immune cell types from bulk RNA-seq data, each based on distinct computational principles. TIMER applies a constrained least square fitting approach to estimate six major immune populations across TCGA cancers. CIBERSORT and CIBERSORT-ABS use support vector regression with reference gene signatures to deconvolute immune-cell proportions, while QUANTISEQ estimates fractions of ten immune-cell types based on normalized expression levels. XCELL employs a gene set enrichment method to compute enrichment scores for a wide range of immune and stromal cell types. MCPCOUNTER quantifies immune and stromal populations using specific marker-gene expression. EPIC estimates both immune and cancer cell fractions, and TIDE (Tumor Immune Dysfunction and Exclusion) models mechanisms of immune evasion to predict infiltration and immune response potential.

Spearman correlation coefficients were calculated between GPAA1 expression and the abundance of each immune cell type inferred by these algorithms. The results were visualized as heatmaps, where darker colors represent stronger positive or negative correlations. In addition, we assessed three immune-related scores in a pan-cancer context using the TCGAplot R package: the Immune Score, Stromal Score, and ESTIMATE Score. These scores were computed based on the ESTIMATE algorithm, which infers the proportion of immune and stromal components in the tumor microenvironment from gene expression data. The Immune Score reflects the level of immune-cell infiltration, the Stromal Score indicates stromal content, and the ESTIMATE Score represents overall non-tumor cell infiltration, inversely reflecting tumor purity. Correlations between GPAA1 expression and these scores were visualized using heatmaps, with * indicating statistically significant associations.

### 2.6 Pan-cancer tumor mutation scoring

The `cor.test` function in R was employed to calculate the correlation between GPAA1 expression and scores for Aneuploidy and Homologous Recombination Defects (HRD). The `fmsb` R package was used to visualize these correlations in a pan-cancer context via spider plots. In the spider plots, axes radiate outward from the center, representing the magnitude of Spearman correlation coefficients. ** indicates *p* < 0.01, and * indicates *p* < 0.05.

### 2.7 Analysis of GPAA1 expression and prognosis in colorectal cancer

We conducted an integrated analysis of six colorectal cancer cohorts from TCGA and GEO to compare GPAA1 mRNA expression and to evaluate prognostic relevance. The TCGA-CRC, also referred to as COADREAD, comprises RNA-seq profiles from tumors and matched adjacent normal tissues and includes clinical annotation across stages I to IV. GSE71187 encompasses specimens from multiple developmental and pathological stages, including embryonic colons (3–22 postovulatory weeks), normal colorectal mucosae, adenomas, and carcinomas, providing insights into gene expression changes from development to malignancy. GSE25071 compares early-onset (mean age 43 years) and late-onset (mean age 79 years) colorectal cancers, with careful matching of clinicopathological parameters and exclusion of hereditary syndromes. GSE39582 is a large-scale cohort (443 tumors, 19 normals) used to define six molecular subtypes of colon cancer associated with distinct biological processes and survival outcomes. GSE41258 includes primary adenocarcinomas, adenomas, metastases, and matched normal mucosae collected at Memorial Sloan Kettering Cancer Center between 1992 and 2004, enabling investigation of tumor progression. GSE87211 consists of 243 rectal cancer samples profiled by expression microarrays and germline Single Nucleotide Polymorphism(SNP) arrays, linking colorectal cancer risk loci (8q24, 18q21, 20q13) to prognosis and chemoradiotherapy resistance. Collectively, these datasets provide a multifaceted resource spanning colorectal development, age-of-onset differences, molecular subtypes, tumor evolution, and treatment response, and used the KM curve of the Log-rank algorithm to associate GPAA1 expression with clinical prognosis (9). Representative immunohistochemistry images for colorectal cancer and normal colon were retrieved from the Human Protein Atlas to provide qualitative validation of GPAA1 protein expression.

### 2.8 Analysis of GPAA1 expression and immune microenvironment in colorectal cancer

Based on the median expression level of GPAA1, samples from the TCGA-CRC cohort were stratified into high- and low-expression groups. The abundance of tumor-infiltrating immune cells was assessed using eight distinct computational algorithms, with heatmaps visualizing infiltration differences between GPAA1 expression groups. To further investigate the association between GPAA1 and the colorectal cancer immune microenvironment, we integrated single-cell RNA sequencing (scRNA-seq) data from the GSE166555 dataset (obtained via the TISCH2 database) and spatial transcriptomic data from 10X Genomics.

### 2.9 Pathway enrichment analysis in CRC

We used the GPAA1 expression in the TCGA database to classify CRC patients into two groups of high and low expression for differential analysis, and the gene names and log2FC values from the differential analysis results were used for inputting into the GSEA enrichment analysis, and then the GO and KEGG enrichment analyses were carried out, which visualized and demonstrated the top five pathways in terms of absolute values of NES.

### 2.10 Cell culture

The human cell lines used in this study included the embryonic kidney epithelial cell line HEK-293T (commonly employed for transfection and protein expression studies) and two CRC cell lines (HCT116 and LOVO). All cell lines were routinely cultured in DMEM high-glucose medium (provided by Thermo Fisher Scientific, MA, USA) supplemented with 10% fetal bovine serum (FBS) and 1% penicillin-streptomycin dual-antibiotic solution. This culture system provided essential nutrients, growth factors, and an aseptic environment for cell growth. All media and supplementary reagents (including trypsin digestion solution and PBS buffer) were purchased from the same supplier to ensure reagent quality consistency and experimental reproducibility. Cell culture was maintained under standard conditions at 37 °C, 5% CO_2_, and saturated humidity. Cell morphology, adherence, and signs of contamination were monitored daily using an inverted microscope.

### 2.11 Plasmid construction

The GPAA1 expression plasmid was constructed by synthesizing the GPAA1 coding sequence and inserting it into the pLVX-Blast vector. Thereafter, the recombinant plasmid was transfected into CRC cells using Lipofectamine 3000 reagent (Thermo Fisher Scientific, MA, USA) in strict accordance with the manufacturer’s guidelines.

### 2.12 Construction of stable expression cell lines

For lentiviral packaging, HEK-293T cells were co-transfected with pLenti-CMV-Flag-Blast (Addgene, MA, USA)—harboring the cDNA sequence of the target gene—along with the packaging plasmids psPAX2 (Addgene, MA, USA) and PMD2.G (Addgene, MA, USA) at a mass ratio of 4:3:2. The viral supernatant was harvested 48 hours post-transfection and passed through a 0.45 μm filter to eliminate cellular debris. Subsequently, HCT116 and LOVO cells were transduced with the recombinant lentiviruses expressing the target gene. To establish stable expression, blasticidin S (Invitrogen, CA, USA) was applied at a concentration of 2 µg/mL for selection two days after infection.

### 2.13 Western blotting

Cells were lysed using RIPA lysis buffer supplemented with freshly added 1× protease inhibitor cocktail. The lysates were centrifuged at 12,000 × g for 15 minutes at 4 °C to remove insoluble debris. Protein concentration in the supernatant was determined using a BCA Protein Assay Kit (Beyotime, Shanghai, China). Equal amounts of protein were separated by SDS-PAGE and electrophoretically transferred to 0.45 μm polyvinylidene difluoride (PVDF) membranes (MilliporeSigma, MA, USA). After transfer, the membranes were blocked with 5% non-fat milk for 1 h at room temperature and then incubated at 4 °C overnight with the following primary antibodies: anti-GPAA1 (1:2000, Proteintech, 10104-1-AP), anti-Flag (1:20,000, Proteintech, 66008-4-Ig), and anti-GAPDH (1:20,000, Proteintech, 10494-1-AP). Following primary antibody incubation, the membranes were washed and incubated with appropriate horseradish peroxidase (HRP)-conjugated secondary antibodies for 1 hour at room temperature. Protein bands were visualized using an enhanced chemiluminescence (ECL) substrate and imaged with a chemiluminescence detection system. Band intensities were quantified using ImageJ software.

### 2.14 Cell viability assay and colony formation assay

#### 2.14.1 Cell viability assay

For cell viability assessment, HCT116 and LOVO cells transfected with GPAA1 were harvested 48 hours post-transfection and seeded into 96-well plates at a density of 1,000 cells per well. At the indicated time points (0, 24, 48, 72, and 96 hours after cell attachment), 10 μL of CCK-8 reagent (Cell Counting Kit-8; Beyotime, Shanghai, China) was added to each well. Following incubation at 37 °C for 2 hours, the absorbance of each well was measured at 450 nm using a microplate reader.

#### 2.14.2 Colony formation assay

In the colony formation experiment, 1,000 cells (or 400 cells for siRNA-transfected groups) were plated per well in 6-well plates, with three replicates for each experimental condition. After 14 days of culture, cells were fixed with 4% paraformaldehyde and stained with crystal violet. The number of colonies was quantified, and statistical analysis was performed using ImageJ software.

### 2.15 Transwell migration and invasion assays

Cell migration and invasion were assessed using 8.0 μm pore-size Transwell chambers (Corning, NY, USA). For the invasion assay, chambers were pre-coated with Matrigel matrix (BD Biosciences, USA) on the membrane’s upper surface. Briefly, cells (5 × 10^4^) in serum-free medium were seeded into the upper chamber, while the lower chamber contained medium with 20% FBS as a chemoattractant. After 48 hours of incubation, cells that migrated or invaded to the underside of the membrane were fixed with 4% paraformaldehyde and stained with crystal violet (Solarbio Technology, Beijing, China). The number of stained cells was counted to evaluate migratory and invasive capacity.

### 2.16 Statistical analysis

All quantitative data were expressed as mean ± SEM from at least three independent experiments. Statistical comparisons between two groups were performed using Student’s t-test, while multiple-group comparisons were analyzed by one-way ANOVA followed by Tukey’s *post-hoc* test (for normally distributed data) or Kruskal-Wallis test (for non-parametric data), using GraphPad Prism 9.0 (GraphPad Software, USA). *p* < 0.05 was considered statistically significant.

## 3 Results

### 3.1 GPAA1 expression patterns, immune subtype associations, and pathway enrichment characteristics across pan-cancers

**Figure 2:**
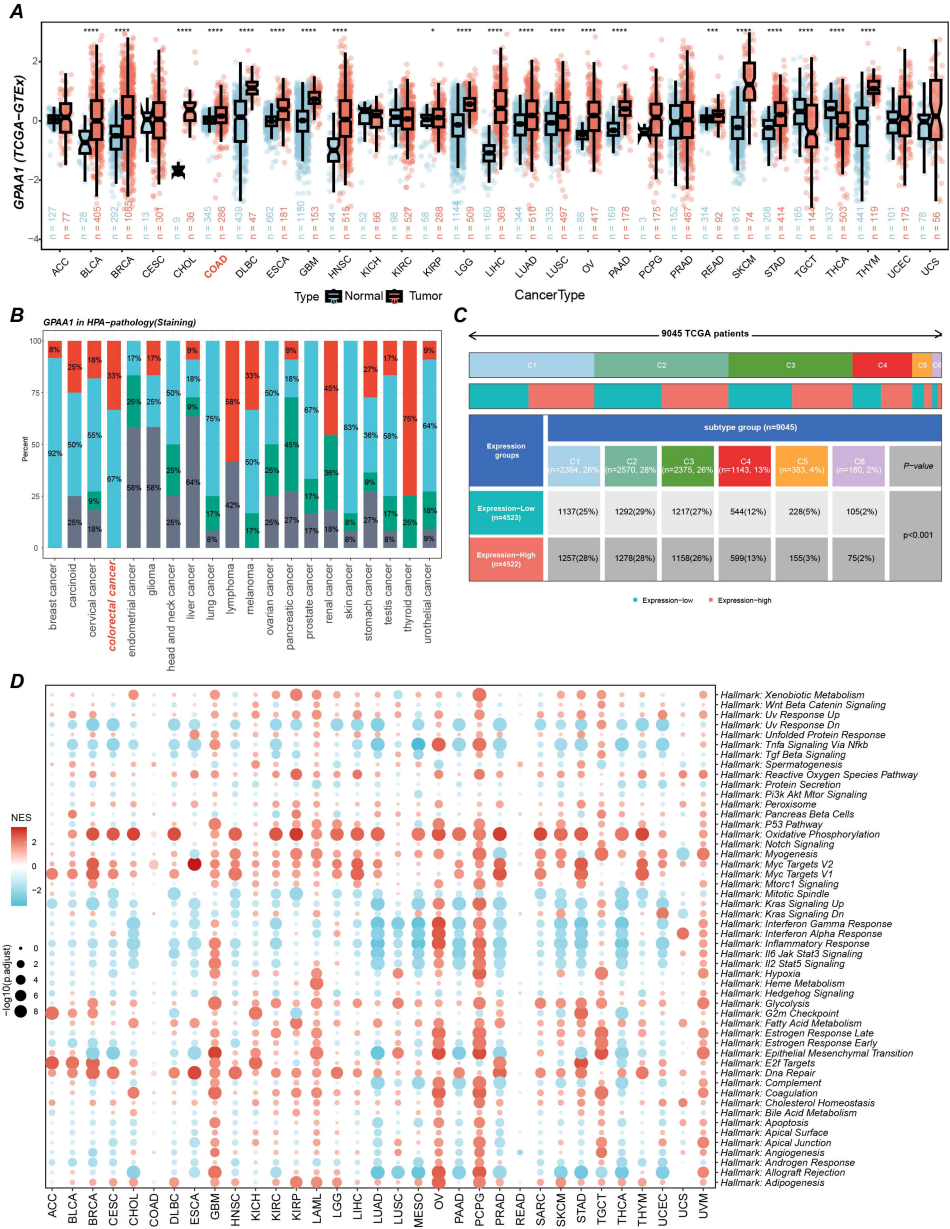
Pan-cancer GPAA1 expression, immune infiltration, and pathway analyses. **(A)** Differences in GPAA1 mRNA expression in the TCGA + GTEx pan-cancer cohort. **(B)** GPAA1 protein staining levels in different cancer types from the HPA pathology dataset. Colors represent staining intensity: red = high, light blue = medium, green = low, gray = not detected. Percentages indicate the proportion of samples in each category. **(C)** Immune-subtype composition differences between GPAA1-high and GPAA1-low groups in the pan-cancer cohort; colors indicate immune subtypes: C1 (wound healing), C2 (IFN-γ dominant), C3 (inflammatory), C4 (lymphocyte-depleted), C5 (immunologically quiet), C6 (TGF-β dominant). **(D)** Hallmark pathway enrichment analysis of GPAA1 in the pan-cancer cohort. * p <0.05, *** p <0.001, **** p <0.0001.

A comprehensive pan-cancer investigation of GPAA1 expression revealed consistently elevated mRNA levels across multiple malignancies, with CRC exhibiting particularly prominent upregulation *(p* < 0.05) upon integration of TCGA and GTEx datasets (**Figure 2A**). This marked overexpression suggests GPAA1’s potential involvement in tumorigenic processes. Immunohistochemical validation via the Human Protein Atlas (HPA) database demonstrated intermediate to high GPAA1 protein expression in most cancer types (**Figure 2B**). Notably, CRC specimens showed significantly greater GPAA1 expression compared to normal tissues, consistent with transcriptional profiling data. Analysis of immune subtype-specific expression patterns indicated substantial elevation of GPAA1 in the C1 (wound healing) subtype (chi-square test, *p* < 0.05; **Figure 2C**), which is characterized by angiogenic gene signatures, enhanced proliferation, and Th2-skewed immune infiltration, and frequently observed in CRC and lung squamous cell carcinoma. These observations imply GPAA1’s role in fostering a tumor-permissive microenvironment through immune subtype modulation. Gene Set Enrichment Analysis (GSEA) additionally revealed activated pathways related to cellular proliferation and metabolic processes in GPAA1-high populations (**Figure 2D**), supporting GPAA1’s function in promoting oncogenesis through core biological mechanisms.

### 3.2 GPAA1 promotes cold tumor phenotype transformation to attenuate Anti-Tumor immune response

**Figure 3:**
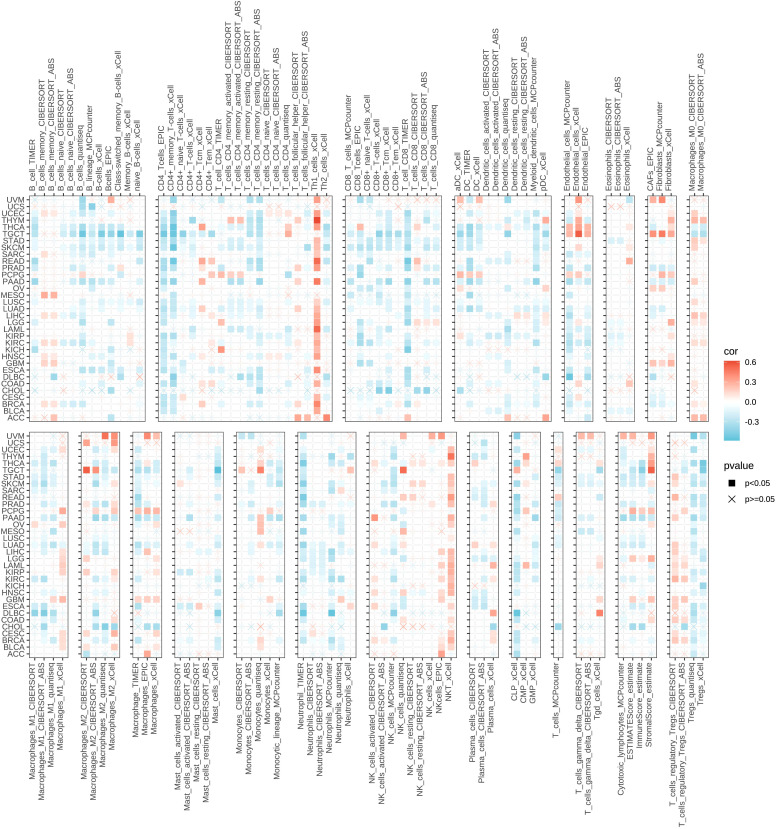
Analysis of GPAA1 expression and the immune microenvironment in pan-cancer.

To further elucidate the extensive impacts of GPAA1 on the tumor immune microenvironment, this research employed immune infiltration analysis relying on multiple algorithms to investigate the relationship between GPAA1 expression and the pan-cancer immune microenvironment. A comprehensive analysis utilizing the TIMER database and eight immune infiltration algorithms (**Figure 3**) indicated that elevated GPAA1 expression was inversely correlated with the degree of tumor immune infiltration. Specifically, the infiltration levels of adaptive immune cell subsets, including CD4 ^+^ T cells, CD8 ^+^ T cells, and memory B cells, were significantly lowered in the group with high GPAA1 expression. Innate immune cells, such as M1 macrophages and neutrophils, also exhibited a general decrease in infiltration. Remarkably, this decline in immune infiltration was especially noticeable in solid tumors such as CRC. These results imply that GPAA1 might promote the tumor microenvironment to transform into a cold tumor phenotype, thus impairing the host’s anti-tumor immune response.

### 3.3 High expression of GPAA1 is significantly associated with genomic instability in CRC

**Figure 4:**
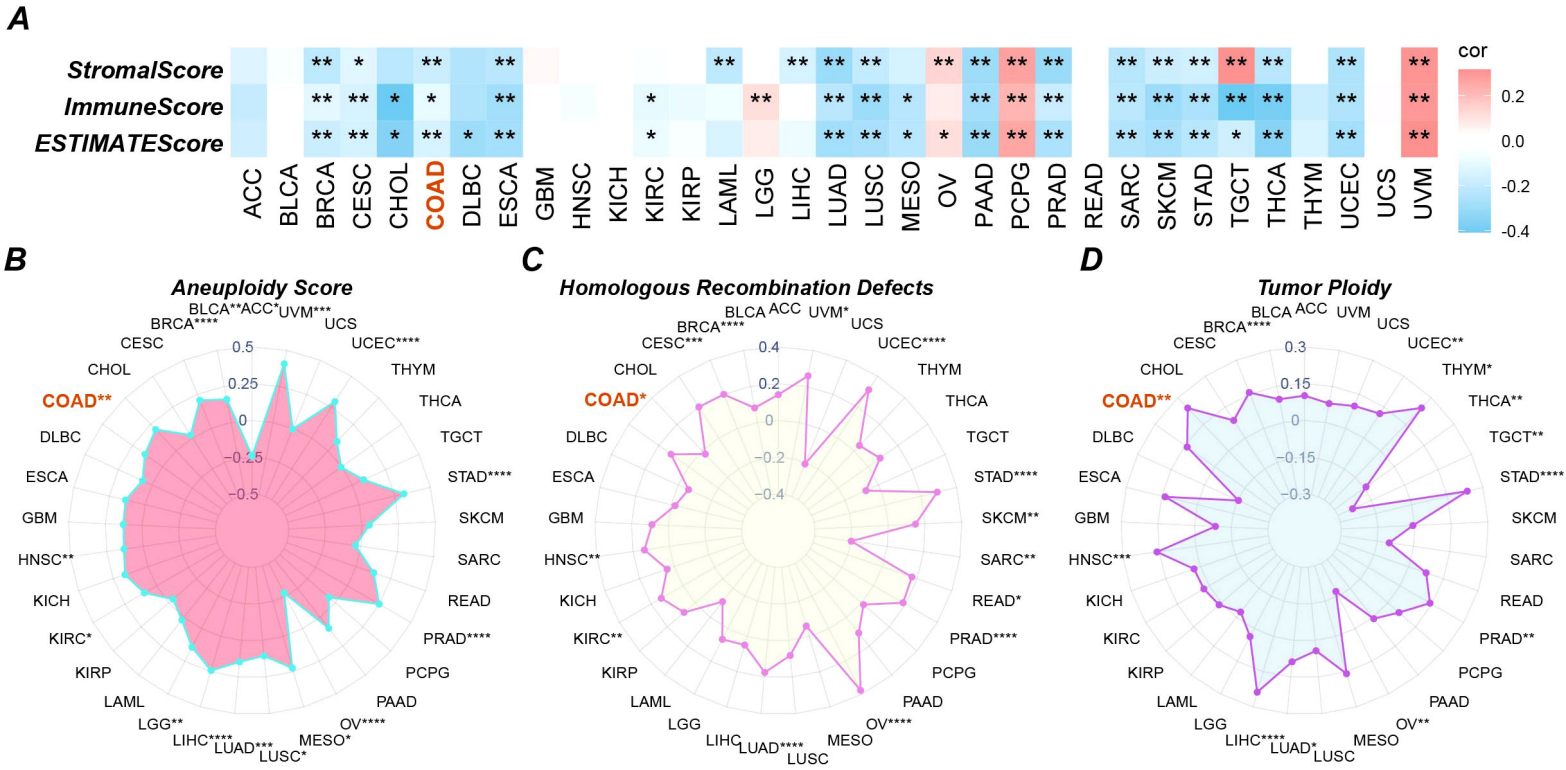
Pan-cancer immune infiltration and gene mutation scores. **(A)** three immune infiltration scores; **(B-D)** three gene mutation scores. * p <0.05, ** p <0.01.

Aneuploidy reflects chromosomal numerical abnormalities, often leading to genomic instability and aggressive tumor behavior. HRD indicates impaired DNA repair capacity, which can confer sensitivity to PARP inhibitors. Tumor Ploidy describes the number of chromosome sets in a cell, with abnormalities suggesting dysregulated cell division. To further validate the immune regulatory role of GPAA1 and its association with genomic instability, analyses of immune scores and genetic mutations were conducted. Regarding immune scoring, analysis using the TCGAplot package (**Figure 4A**) revealed that high GPAA1 expression was significantly negatively correlated with ImmuneScore, StromalScore, and ESTIMATEScore. This finding further confirms GPAA1’s role in promoting a cold tumor microenvironment. In terms of genetic mutation characteristics, GPAA1 expression was positively correlated with aneuploidy (**Figure 4B**), HRD (**Figure 4C**), and tumor ploidy (**Figure 4D**) (*p* < 0.05), with these associations being particularly pronounced in CRC. The positive correlation between GPAA1 expression and these markers suggests that GPAA1 may promote CRC progression by driving genomic instability, thereby accelerating tumor evolution and therapeutic resistance. For example, HRD may increase tumor sensitivity to PARP inhibitors, offering a potential therapeutic target for CRC.

### 3.4 GPAA1 is highly expressed in CRC and significantly shortens overall survival in CRC patients

**Figure 5:**
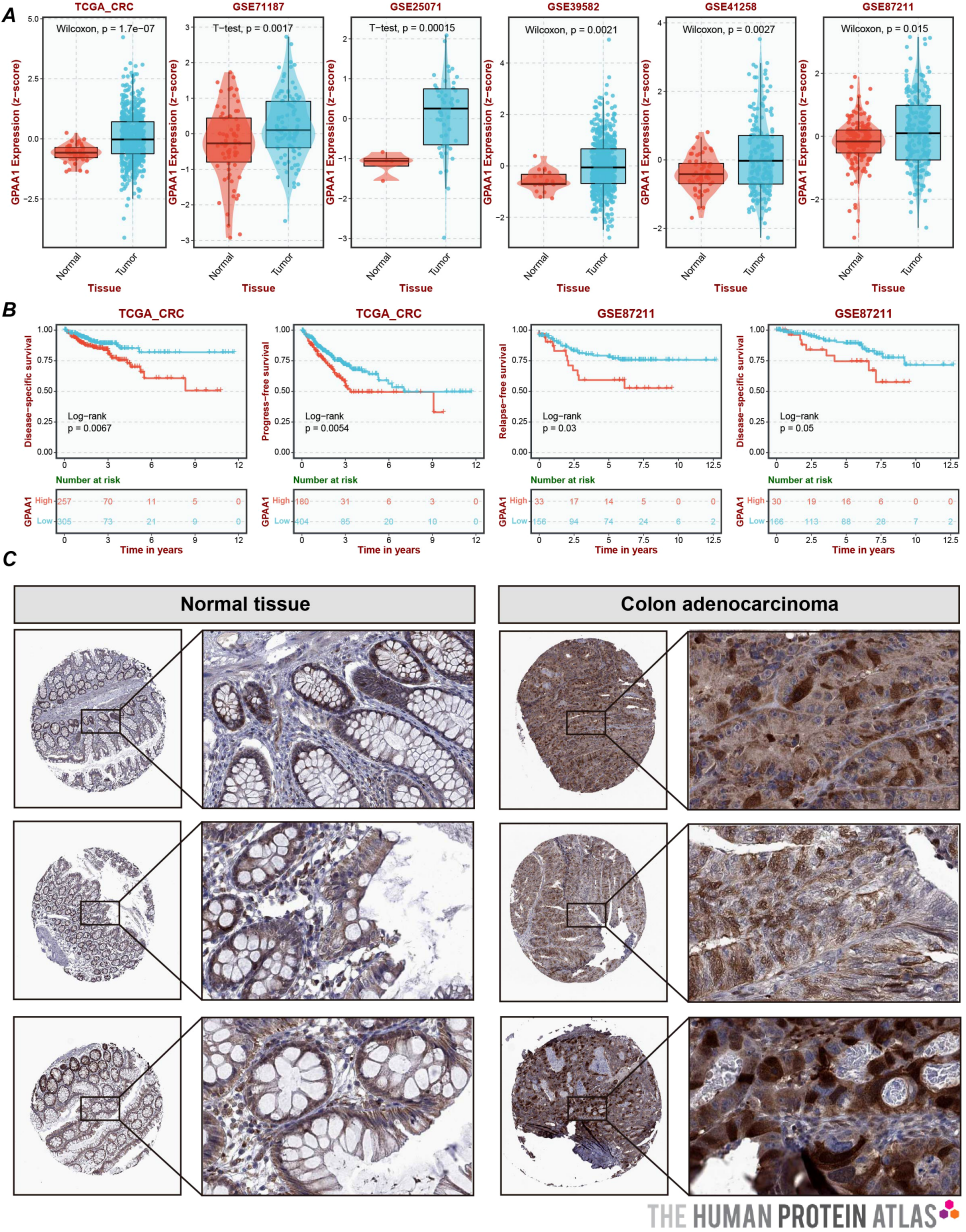
Expression and prognostic analysis of GPAA1 in CRC. **(A)** Joint analysis of GPAA1 mRNA expression differences by TCGA and GEO; **(B)** Kaplan-Meier survival curves depicting the overall survival of CRC patients stratified by GPAA1 expression levels. Patients were divided into high-expression and low-expression groups based on the median GPAA1 mRNA level in the combined TCGA and GEO cohorts. The log-rank test was used to assess the difference in survival between the two groups. The p-value is indicated on the graph; **(C)** HPA-derived IHC sections demonstrating the differences in GPAA1 protein expression between normal and cancerous tissues.

Systematic verification was conducted on the expression differences and clinical prognostic significance of GPAA1 in CRC. To verify expression discrepancies (**Figure 5A**), a combined analysis of the TCGA-CRC dataset and five Gene Expression Omnibus (GEO) datasets showed that the mRNA expression of GPAA1 in CRC tumor tissues was significantly higher compared to normal tissues (*p* < 0.05). IHC results from the HPA database (**Figure 5C**) further validated that GPAA1 protein exhibited moderate to high expression in CRC tissues, matching the previously observed mRNA expression pattern. Regarding the analysis of prognostic relevance, Kaplan-Meier survival curves (**Figure 5B**) indicated that CRC patients in the high-GPAA1 expression group experienced notably reduced overall survival (*p* < 0.05). Collectively, these findings imply that GPAA1 could serve as an independent biomarker predicting poor prognosis in CRC.

### 3.5 GPAA1 modulates the local immune microenvironment in CRC

**Figure 6:**
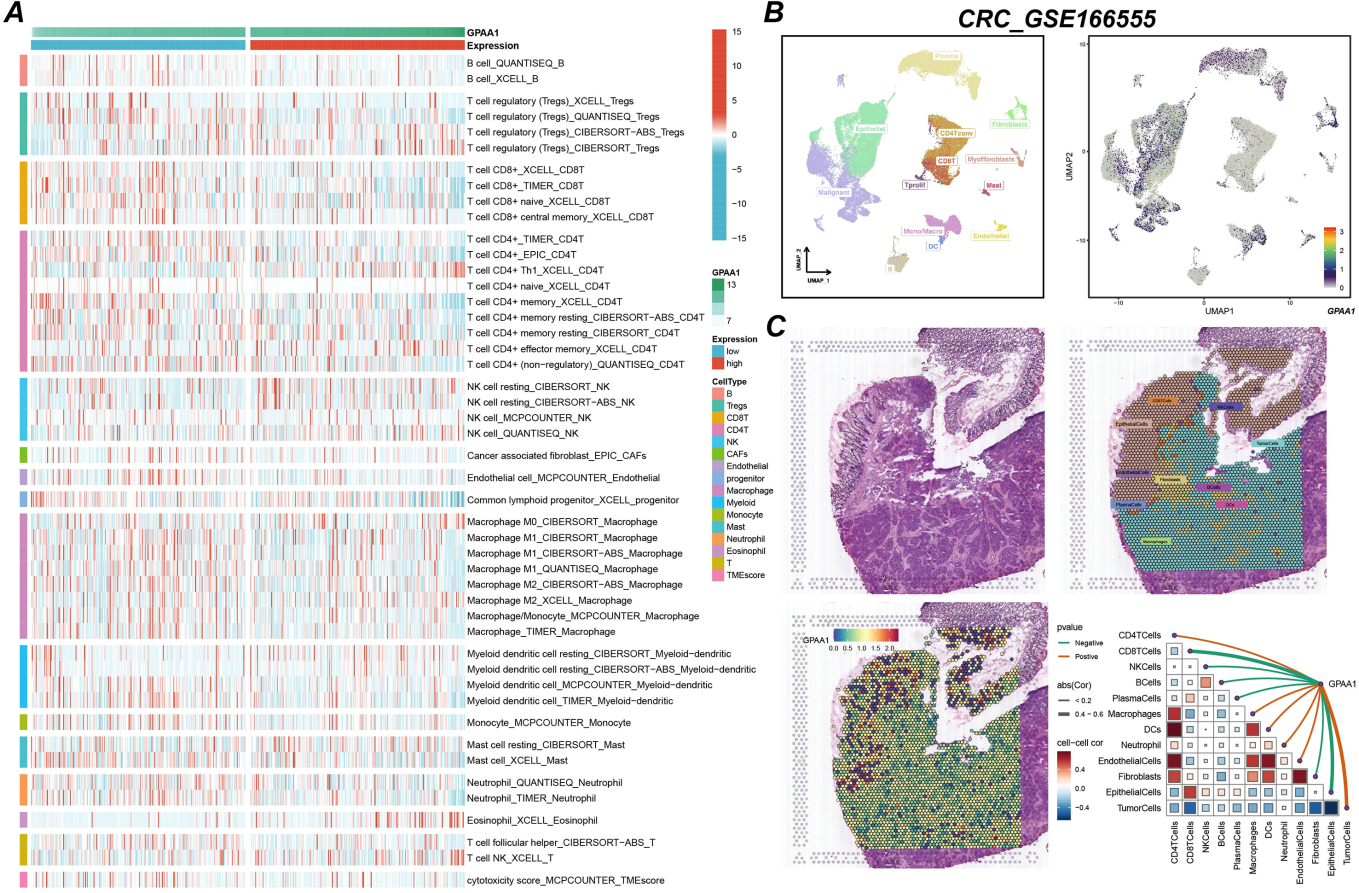
Analysis of GPAA1 immune infiltration in CRC. **(A)** Differences in immune cell infiltration between high and low GPAA1 expression groups; **(B)** single-cell sequencing analysis of cell subtypes with high GPAA1 expression; **(C)** spatial transcriptome analysis of GPAA1 expression distribution and its association with immune infiltration.

This study utilized single-cell and spatial transcriptomic technologies to gain an in-depth understanding of GPAA1’s role in the local immune microenvironment of CRC. Analysis of immune cell infiltration differences (**Figure 6A**) revealed that in the high-expression group, the infiltration abundance of anti-tumor immune cells (such as CD8+ naïve T cells and memory B cells) was significantly reduced, while the infiltration of pro-cancer cells (such as M2 macrophages) increased (*p* < 0.05). This suggests that GPAA1 may promote tumor progression by altering the immune cell composition. Single-cell sequencing results (**Figure 6B**) indicated that high GPAA1 expression was primarily enriched in tumor cells and cancer-associated fibroblasts (CAFs), suggesting these cells might be key carriers through which GPAA1 exerts its effects. Spatial transcriptomics (**Figure 6C**) further confirmed that GPAA1 expression hotspot regions were negatively correlated with the spatial distribution of CD8+ T cells and NK cells, implying a potential mechanism of suppressing immune infiltration by regulating spatial cell arrangement. Additionally, a heatmap (**Figure 6C**) showed significant negative correlations with CD8+ T cells and NK cells, and a positive correlation with M2 macrophages. These results provide direct cellular-level evidence for GPAA1 promoting an immunosuppressive cold tumor phenotype in CRC.

### 3.6 Differential gene and functional pathway enrichment analysis of GPAA1 in CRC

**Figure 7:**
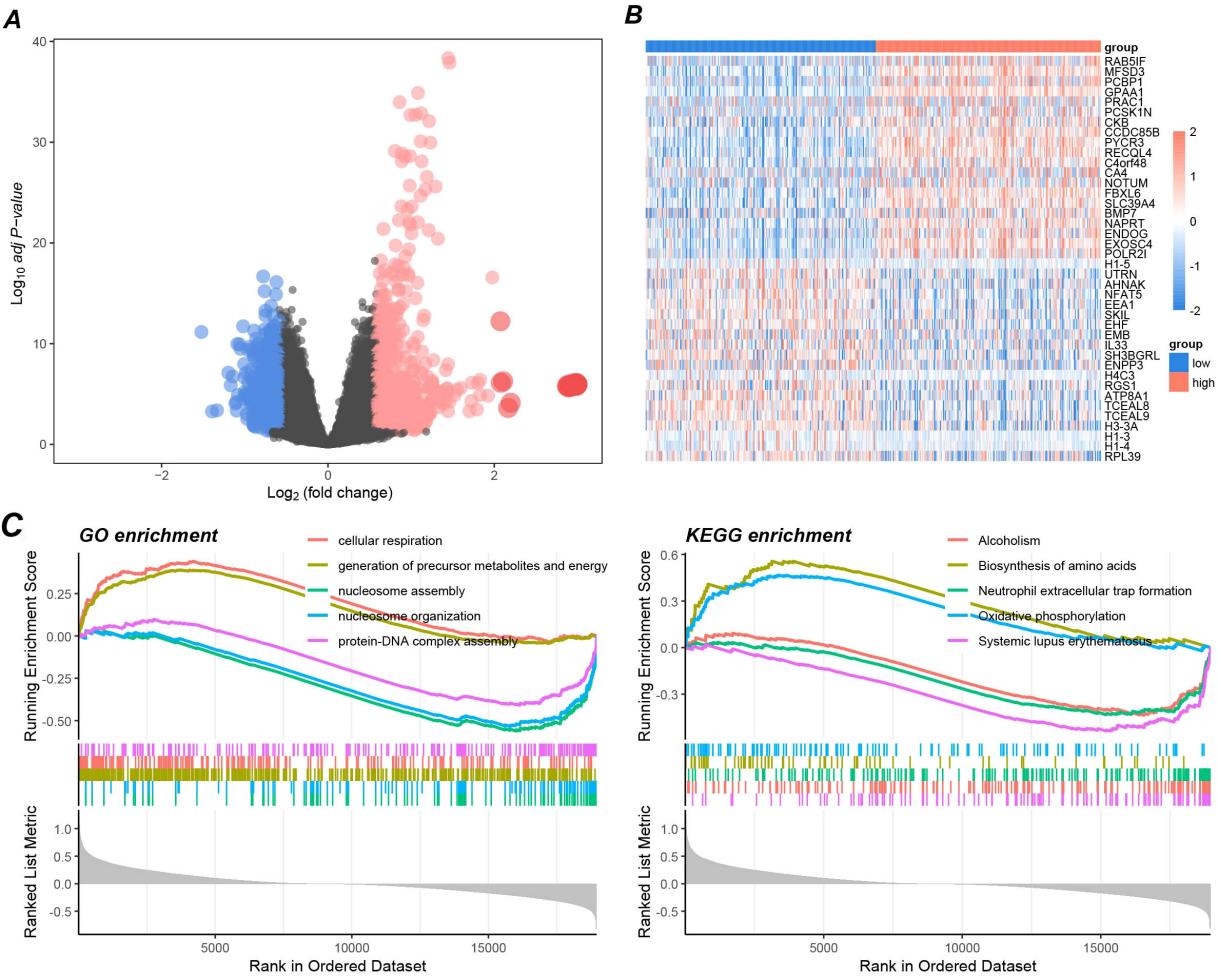
Pathway enrichment analysis of GPAA1 in CRC. **(A)** Differential gene volcano map between high and low GPAA1 expression groups; **(B)** Top differential gene display heatmap; **(C)** GSEA-driven GO and KEGG enrichment analysis.

To further elucidate the potential molecular pathways through which GPAA1 functions in CRC, this study conducted differential gene and functional enrichment analyses. Differential gene analysis results (**Figure 7A**) showed that after grouping based on the median gene expression, over 2000 genes were significantly upregulated (Log2FC > 1) in the GPAA1 high-expression group. These genes are primarily involved in biological processes related to metabolism and immune regulation (**Figure 7B**). GSEA results revealed that GO analysis (**Figure 7C**) was mainly enriched in processes such as “cellular respiration” and “generation of precursor metabolites and energy,” suggesting that GPAA1 may enhance the metabolic activity of tumor cells to promote their proliferation. KEGG analysis (**Figure 7C**) enriched pathways such as “Oxidative phosphorylation” and “Neutrophil extracellular trap formation.” This finding further reveals that GPAA1 is associated with enhanced energy metabolism and may potentially suppress immune response by inhibiting neutrophil function.

### 3.7 Enhanced GPAA1 expression promotes proliferation and clonogenicity in CRC cells

**Figure 8:**
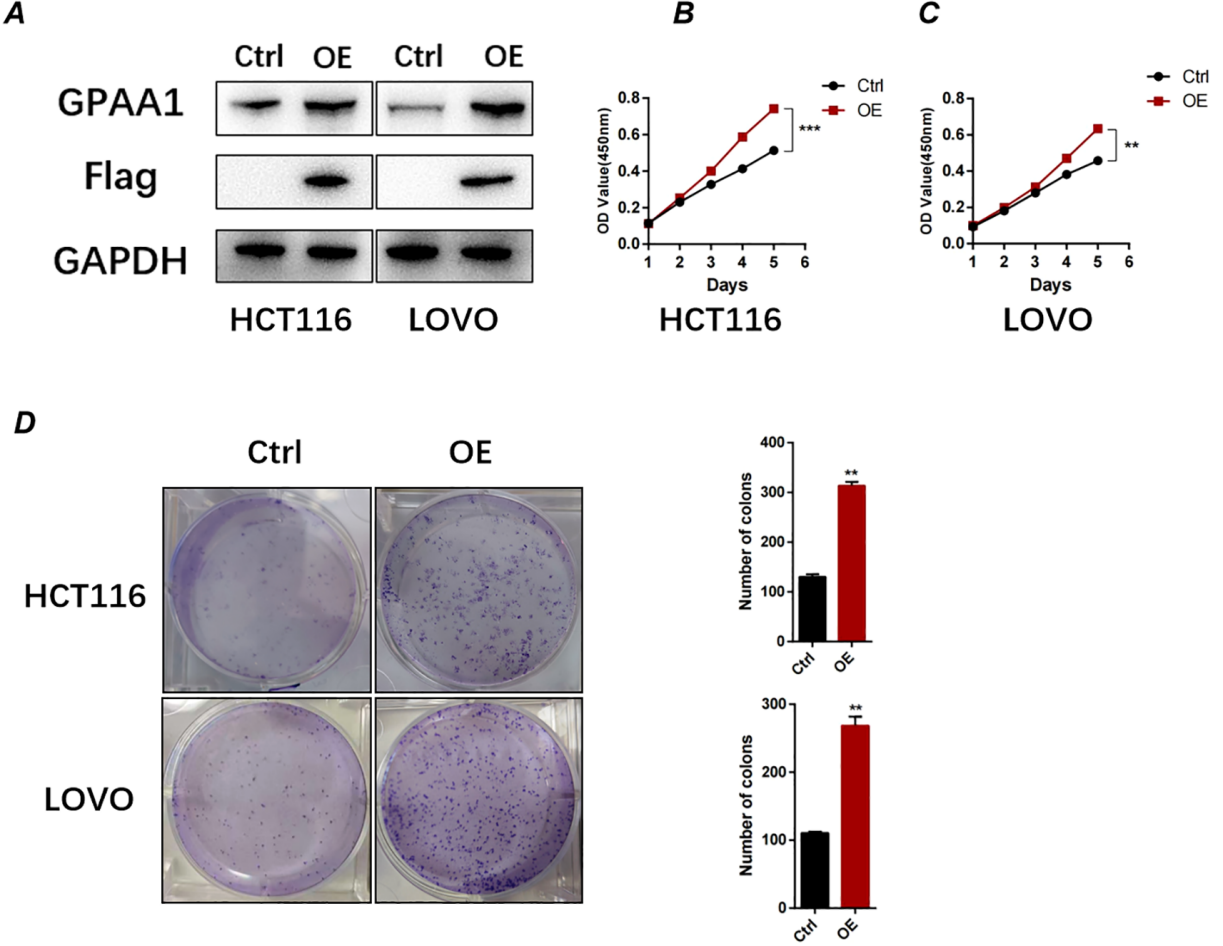
GPAA1 overexpression promotes proliferation and clonogenicity in colorectal cancer cells in vitro. **(A)** Western blot analysis confirming GPAA1 overexpression in HCT116 and LOVO cells transfected with GPAA1 plasmid (OE) compared to empty vector control (Ctrl). Flag tag indicates exogenous GPAA1 expression; GAPDH serves as loading control.**(B, C)** Cell proliferation measured by CCK-8 assay in HCT116 **(B)** and LOVO **(C)** cells over 6 days. GPAA1 overexpression significantly enhanced proliferation rates (**P<0.01, ***P<0.001). **(D)** Colony formation assays (left) and quantitative analysis (right) demonstrating that GPAA1 overexpression increased both the number and size of colonies in both cell lines (**P<0.01).

To further investigate the functional role of GPAA1 in CRC, we overexpressed GPAA1 in HCT116 and LOVO cell lines using a lentiviral vector carrying the GPAA1 cDNA sequence. Successful overexpression was confirmed at the protein level by Western blot analysis (**Figure 8A**). Functional assays revealed that GPAA1 overexpression significantly enhanced cellular proliferation, as measured by CCK-8 assay (**Figures 8B, C**), and increased clonogenic survival in colony formation assays (**Figure 8D**), indicating a promotive role of GPAA1 in CRC cell growth and clonogenicity.

### 3.8 GPAA1 enhances migratory and invasive capacities in CRC cells

**Figure 9:**
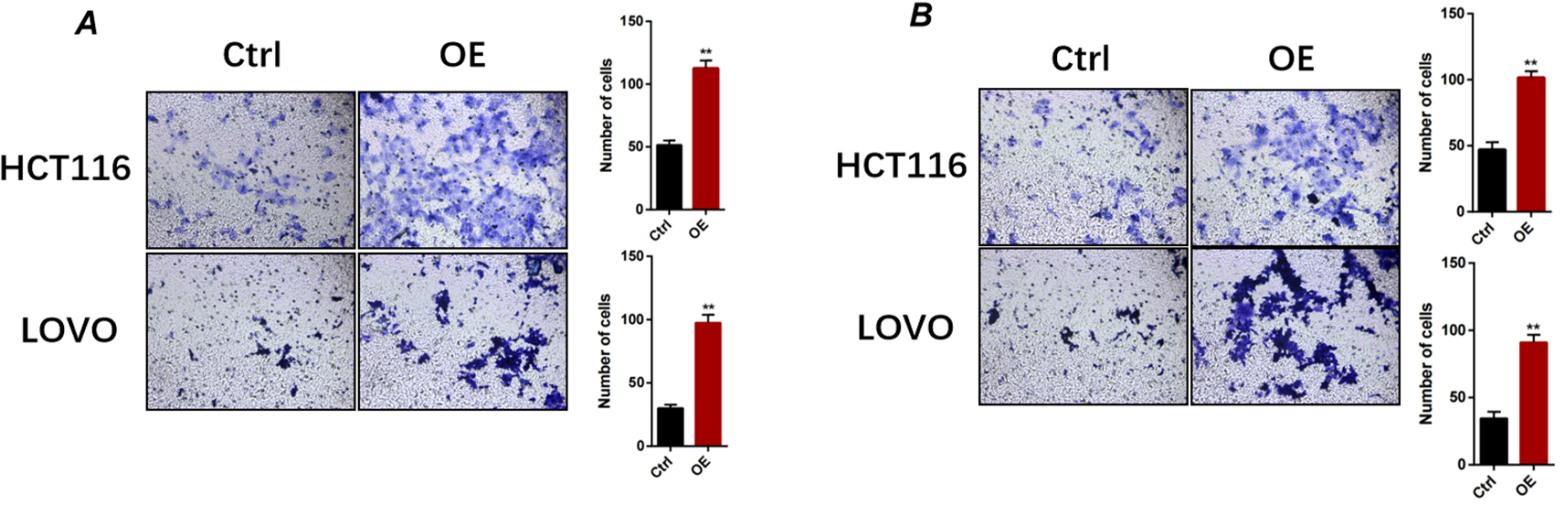
GPAA1 overexpression enhances migratory and invasive capacities of colorectal cancer cells *in vitro*. **(A)** Representative images (left) and quantitative analysis (right) of migrated HCT116 and LOVO cells under control (Ctrl) and GPAA1-overexpressing (OE) conditions. **(B)** Representative images (left) and quantitative analysis (right) of invaded HCT116 and LOVO cells. ** p <0.01.

Given that GPAA1 expression was correlated with lymph node metastasis in CRC patients, we further explored its function in regulating the migration and invasion of CRC cells. Transwell assays demonstrated that GPAA1 overexpression significantly boosted the migratory capacity of HCT116 and LOVO cells (**Figure 9A**). Likewise, GPAA1 overexpression also notably enhanced the invasive potential of these two CRC cell lines (**Figure 9B**).

## 4 Discussion

This study systematically investigates the expression patterns, biological functions, and clinical implications of GPAA1 across pan-cancers, with a specific focus on CRC. The integrated multi-omics, single-cell, spatial transcriptomic, and *in vitro* functional data collectively reveal that GPAA1 acts as a pro-tumorigenic factor in CRC, offering novel insights into its role in tumor progression and immunosuppression, as well as potential clinical value.

The pan-cancer analysis revealed that GPAA1 is consistently overexpressed across multiple malignancies, with particularly striking upregulation in CRC. This observation aligns with previous reports suggesting that components of the GPI-anchor biosynthesis pathway may be dysregulated in cancer (4, 10, 11). The functional experiments demonstrated that GPAA1 overexpression enhances proliferation, clonogenicity, migration, and invasion in CRC cell lines. These findings are consistent with the pathway enrichment analyses showing activation of proliferation and metabolism-related pathways in GPAA1-high tumors. The observed metabolic reprogramming, particularly the enrichment of oxidative phosphorylation pathways, suggests that GPAA1 may promote a metabolic state favorable for tumor growth and survival, similar to what has been reported for other cancer-related genes (5, 6, 12, 13).

A particularly significant finding of our study is the association between GPAA1 expression and genomic instability markers, including aneuploidy, HRD, and tumor ploidy. This suggests that GPAA1 may contribute to CRC progression not only through direct effects on tumor cell behavior but also by promoting genomic instability, which could accelerate tumor evolution and therapeutic resistance (14). The positive correlation with HRD is especially noteworthy as it suggests that GPAA1-high tumors might be particularly susceptible to PARP inhibitors, providing a potential therapeutic strategy for this patient subset.

Perhaps the most clinically relevant finding is the demonstration that GPAA1 promotes an immunosuppressive tumor microenvironment characteristic of cold tumors (15, 16). Our multi-algorithm immune infiltration analysis revealed that high GPAA1 expression correlates with reduced infiltration of CD8+ T cells and other anti-tumor immune cells, while increasing pro-tumor M2 macrophages. These findings were further supported by single-cell and spatial transcriptomic analyses, which showed that GPAA1 expression hotspots are spatially segregated from CD8+ T cell and NK cell infiltrates. This spatial organization pattern may contribute to immune evasion by creating physical barriers between tumor cells and immune effectors (17, 18). The association with the C1 wound healing immune subtype, known for its Th2-skewed, pro-angiogenic phenotype, further underscores GPAA1’s role in shaping an immunosuppressive microenvironment. The underlying mechanisms by which GPAA1 exerts its regulatory effects on the immune microenvironment remain to be comprehensively clarified. Our pathway analyses suggest several possibilities, including modulation of neutrophil extracellular trap formation and metabolic reprogramming that could alter immune cell function. The enrichment of oxidative phosphorylation pathways in GPAA1-high tumors might create a metabolically hostile environment for immune cells. The positive correlation between GPAA1 expression and M2 macrophage infiltration suggests a potential regulatory link, but whether GPAA1 directly promotes M2 polarization remains to be confirmed *in vitro*.

Several limitations of this study should be acknowledged. First, while we identified a correlation between GPAA1 and HRD, we did not explore the molecular mechanism linking GPAA1 to homologous recombination. Second, our *in vitro* functional assays were limited to HCT116 and LOVO cell lines—validation in additional CRC cell lines (e.g., SW480) or patient-derived organoids (PDOs) would enhance generalizability. Third, *in vivo* studies using CRC xenograft models are needed to confirm whether GPAA1 inhibition reverses the cold tumor phenotype and suppresses tumor growth. Lastly, the study needs to account for interpatient and clinical phenotypic variability, particularly factors such as treatment, comorbidities, lifestyle, smoking status, age, and gender. As the analysis relies heavily on publicly available datasets, heterogeneity in data processing, sample collection, and clinical annotation may introduce potential biases.

Despite these limitations, our study has important clinical implications. The strong association between GPAA1 expression and cold tumor phenotype suggests that GPAA1 could serve as a biomarker to identify patients less likely to respond to immune checkpoint inhibitors. Conversely, the association with HRD raises the possibility that PARP inhibitors might be effective in GPAA1-high tumors, providing a potential alternative treatment strategy. Future research should focus on (1): Identifying upstream regulators of GPAA1 overexpression in CRC, Such as DNA Methylation or Transcription Factors (2); Testing the efficacy of PARP inhibitors in GPAA1-high CRC xenografts (3); Exploring whether combining GPAA1 silencing with immune checkpoint inhibitors can convert cold tumors to hot tumors and improve therapeutic response.

## 5 Conclusion

In conclusion, our study establishes GPAA1 as a multi-functional oncogenic factor in CRC that promotes tumor progression through direct effects on tumor cell behavior, induction of genomic instability, and creation of an immunosuppressive microenvironment. These findings position GPAA1 as a promising prognostic biomarker and potential therapeutic target in CRC, particularly for tumors with cold immune phenotypes. Further investigation is essential to elucidate the precise molecular mechanisms through which GPAA1 exerts its multifaceted oncogenic effects, and to rigorously evaluate its potential as a therapeutic target in both preclinical models and clinical settings.

## Data Availability

The original contributions presented in the study are included in the article/supplementary material. Further inquiries can be directed to the corresponding author/s.
